# Juvenile idiopathic arthritis of the knee: is contrast needed to score disease activity when using an augmented MRI protocol comprising PD-weighted sequences?

**DOI:** 10.1007/s00330-022-09292-3

**Published:** 2022-12-06

**Authors:** V. D. Vo Chieu, V. Vo Chieu, F. Dressler, N. Kornemann, A. Pfeil, J. Böttcher, F. Streitparth, L. D. Berthold, M. Dohna, D. M. Renz, S. Hellms

**Affiliations:** 1 Institute for Diagnostic and Interventional Radiology, Hannover Medical School, Carl-Neuberg Str. 1, 30625 Hanover, Germany; 2 Institute for Diagnostic and Interventional Radiology, St. Vincenz-Krankenhaus Paderborn, Paderborn, Germany; 3 Pediatric Rheumatology Clinic, Division of Pediatric Pneumology, Allergology and Neonatology, Hannover Medical School, Hanover, Germany; 4 Department of Internal Medicine III, Jena University Hospital, Friedrich-Schiller-University, Jena, Germany; 5 Institute for Diagnostic and Interventional Radiology, Jena University Hospital, Friedrich-Schiller-University of Jena, Jena, Germany; 6 Department of Radiology, University Hospital Munich, Munich, Germany; 7 University Hospital Gießen and Marburg, Clinic for Diagnostic and Interventional Radiology and Pediatric Radiology, Giessen, Germany

**Keywords:** Juvenile idiopathic arthritis, Knee joint, Magnetic resonance imaging, Contrast media

## Abstract

**Objective:**

To compare unenhanced versus enhanced knee joint magnetic resonance imaging (MRI) to assess disease activity of juvenile idiopathic arthritis (JIA).

**Methods:**

Fifty-three knee joint MRI examinations were performed on a 3-Tesla system in 27 patients (age: 11.40 ± 3.61 years; 21 females, 6 males). MRI protocols comprised PD-weighted sequences in addition to the widely used standard protocol. JIA subgroups comprised oligoarticular arthritis (*n* = 16), extended oligoarthritis (*n* = 6), rheumatoid factor-negative polyarticular arthritis (*n* = 3), enthesitis-related arthritis (*n* = 1), and psoriatic arthritis (*n* = 1). MR images were retrospectively analyzed by 3 experienced radiologists in two readings, using JAMRIS (juvenile arthritis MRI scoring) system and a modified IPSG (international prophylaxis study group) classification. In the first reading session, only unenhanced MR images were evaluated. In a second reading session, all images before and after contrast medium application were included. In order to avoid bias, an interval of at least 2 weeks was set between the two readings. The clinical JADAS10 (juvenile arthritis disease activity score) was calculated including clinical assessment and laboratory workup and correlated with MRI scores. Statistical analysis comprised Pearson’s correlation for correlating two scoring results of unenhanced and the enhanced MRI, intra-class correlation coefficient (ICC) for inter- and intra-reader agreement. Diagnostic accuracy was calculated using ROC (receiver operating characteristics) curve analysis.

**Results:**

Inter-reader agreement determined by ICC for unenhanced and enhanced MRI scores for IPSG was moderate (0.65, 95% CI 0.51–0.76, and 0.62, 95% CI 0.48–0.75) and high for JAMRIS (0.83, 95% CI 0.75–0.89, and 0.82, 95% CI 0.74–0.89). Intra-reader agreement was good to very good for JAMRIS (0.85 95% CI 0.81–0.88, 0.87 95% CI 0.83–0.89 and 0.96 95% CI 0.92–0.98) and IPSG (0.76 95% CI 0.62–0.86, 0.86 95% CI 0.77–0.92 and 0.92 95% CI 0.86–0.96). Scores of unenhanced MRI correlated with contrast-enhanced MRI: JAMRIS (*r* = 0.97, *R*^2^ = 0.93, *p* < 0.01), modified IPSG (*r* = 0.95, *R*^2^ = 0.91, *p* < 0.01). When using JADAS10 as a reference standard, moderate accuracy for both unenhanced and enhanced MRI scores was noted: JAMRIS (AUC = 0.68, 95% CI 0.51–0.85, and AUC = 0.66, 95% 0.49–0.82), IPSG score (AUC = 0.68, 95% 0.50–0.86, and AUC = 0.61, 95% 0.41–0.81).

**Conclusions:**

Our results suggest that contrast agent application could be omitted in JIA patients with an augmented knee MRI protocol comprising PD-weighted sequence.

**Key Points:**

*• Unenhanced MRI can detect disease activity of the knee joint in patients with JIA with equally high accuracy compared to contrast-enhanced MRI.*

*• The intra- and inter-reader agreement was high for unenhanced and enhanced MRI JAMRIS scores, which indicate relatively good applicability of the scoring system, even for less experienced readers.*

*• When using the clinical JADAS10 as a reference standard for the detection of disease activity, moderate accuracy for both unenhanced and enhanced MRI scores, both JAMRIS and IPSG, was noted, which might be caused by the fact that the majority of patients had either no or minimal clinical disease activity.*

**Supplementary Information:**

The online version contains supplementary material available at 10.1007/s00330-022-09292-3.

## Introduction

Juvenile idiopathic arthritis (JIA) is a heterogeneous group of inflammatory musculoskeletal diseases comprising all forms of arthritis that begin before the age of 16 years, persist for more than 6 weeks, and are of unknown etiology and pathophysiology [1]. The yearly incidence in developed countries is specified as 2–20 cases/100,000 children [2] which makes it one of the most common chronic inflammatory diseases in pediatric patients. Inflammation in the affected joints leads to synovial proliferation with consecutive secretion of synovial fluid and synovial hypertrophy [3]. This again can lead to cartilage lesions and bone erosions, resulting in pain and disability [4, 5]. Minimizing time to treatment after onset of synovial inflammation is reported to improve long-term outcomes [6–8]. Magnetic resonance imaging (MRI) and Doppler ultrasound are frequently used to detect inflammation within the joint before destruction occurs as well as for monitoring disease progression and treatment response [9]. Ultrasonography is commonly used to monitor joint effusion and synovial hypertrophy [10]. MRI is considered the most sensitive imaging technique for the evaluation of joint inflammation [11, 12] and is well adapted to monitor disease activity especially in pediatric patients, as it is non-ionizing.

Various MRI scoring systems have been developed to assess disease and to monitor activity on MR imaging including the juvenile arthritis MRI scoring (JAMRIS) system for the knee and the international prophylaxis study group (IPSG) scores [13–16]. In both MRI scoring systems, features such as cartilage lesions and bone involvement (marrow edema and erosions, cysts) are objectified in a point score according to pathological findings; assessment of synovial membrane thickness as a measure of synovitis is yet another crucial point [17].

MRI protocols usually comprise contrast-enhanced sequences for the detection of synovitis, which is reported to be significantly associated with the clinical onset of JIA [18]. However, contrast-enhanced MRI can be challenging.

Intravenous administration of gadolinium-based contrast medium is associated with increased costs and risks [19]. Furthermore, the recently described possible accumulation of gadolinium in deep brain nuclei such as the dentate nucleus and globus pallidus with uncertain long-term consequences leads to stricter indications for the use of contrast agents in MR imaging [20–22]. In addition, examination time should be limited, especially in younger patients as they are more prone to movements, which augment with the duration of the examination [23].

The aim of our study was to compare whether unenhanced MRI, using an augmented protocol comprising PD-weighted sequences, can be used for reliable detection of disease activity in JIA with similar accuracy to contrast-enhanced MRI sequences and may therefore serve as a feasible alternative.

## Material and methods

### Study design and subjects

This study was approved by the local Institutional Review Board (No. 9798_BO_K_2021). This retrospective study involved patients who were diagnosed with JIA and had an MRI of the knee performed within a 5-year time period (between 01/2015 and 01/2020).

All patients were treated and clinically assessed by an experienced pediatric rheumatologist (over 30 years of experience) and received laboratory workup within 4 weeks before or after the MRI examination.

The clinical 10-joint juvenile arthritis disease activity score (JADAS10) was calculated including the following four criteria with a maximum score of 40 points: physician global assessment of disease activity on a linear visual analogue scale (VAS) of 0–10; a subjective patient or parent assessment on a VAS of 0–10; normalized erythrocyte sedimentation rate of 0–10; and active joint count from 0–10, any count over 10 joints yielding the maximum score of 10 [24]. The cutoff value to define inactive disease was a JADAS10 of ≤ 1.4 for oligoarthritis and ≤ 2.7 for polyarthritis according to the updated 2021 definition [25, 26] (Supplement table 1).

**Fig. 1:**
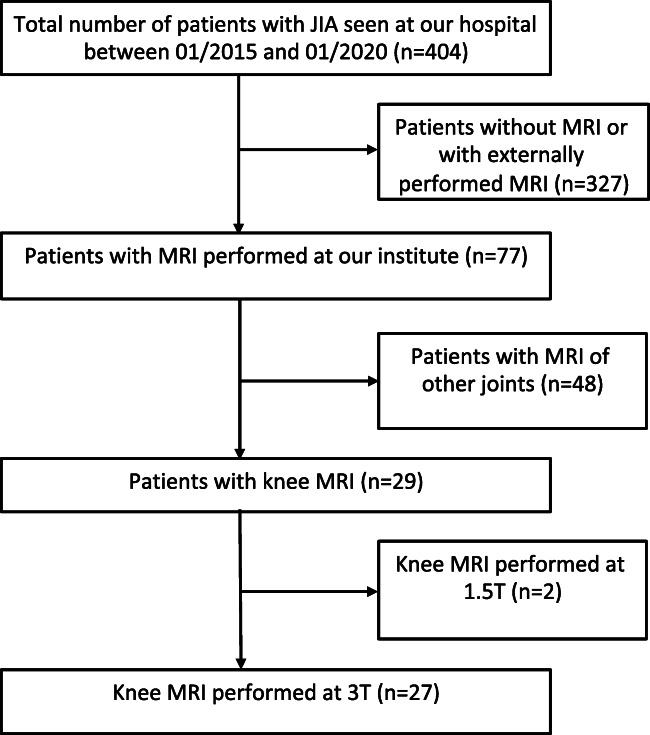
Patient inclusion chart. The total number of patients referred to the Institute for Diagnostic and Interventional Radiology by the Pediatric Rheumatology Clinic (for x-ray, sonography, or demonstration of external images) with confirmed or suspected JIA between 01/2015 and 01/2020 were searched. Only JIA patients with knee MRI performed on our 3-Tesla system (Magnetom Verio) were included in our study

Our study included 27 patients with JIA (female *n* = 21, 77.8%; male *n* = 6, 22.2%). All consecutive contrast-enhanced knee MRI examinations of JIA patients with suspected disease activity that were performed between January 2015 and January 2020 were included in the study. A total of 53 knee joints were examined, with two patients undergoing two or more MRI examinations with an interval of at least 6 months due to suspected disease activity. For inclusion criteria, see Fig. 1. The average age of patients was 11.40 ± 3.61 years at the time of the MRI.

The JIA subgroups included oligoarticular arthritis (*n* = 16), extended oligoarthritis (*n* = 6), rheumatoid factor-negative polyarticular arthritis (*n* = 3), enthesitis-related arthritis (*n* = 1), and psoriatic arthritis (*n* = 1). The average number of affected joints was 4 ± 3 joints.

Every patient involved in the study received medication, including one or two of the following: naproxen (*n* = 19), ibuprofen (*n* =1), methotrexate (*n* = 8), etanercept (*n* = 1), and prednisolone (*n* = 1) (Table 1).

**Table 1:** Patient characteristics

Number of patients	*n* = 27
Gender (female)	*n* = 21 (77.8%)
Number of knees	53
Average age of patients	11.40 ± 3.61 years
JIA subgroups	Oligoarticular arthritis (*n* = 16) Extended oligoarthritis (*n* = 6) Rheumatoid factor-negative polyarticular Arthritis (*n* = 3) Enthesitis-related arthritis (*n* = 1) Psoriatic arthritis (*n* = 1).
Medication	Naproxen (*n* = 19) Ibuprofen (*n* =1) Methotrexate (*n* = 8) Etanercept (*n*=1) Prednisolone (*n* = 1)
Clinically active disease	Only patients with oligoarticular arthritis had active disease (*n* = 8/16): Of these: Minimal disease activity (*n* = 6/8) Moderate disease activity (*n* = 1/8) High disease activity (*n* = 1/8)

### Imaging technique

All MRI examinations were performed on a 3-Tesla system (Magnetom Verio, Siemens Healthineers) using either a 6-channel body coil to simultaneously examine both knees during one single exam or a dedicated 6-channel transmit/receive knee coil to examine one knee. Of a total of 53 knee joints, nine were examined with a knee coil and 44 with a 6-channel body coil. Sequences included: proton-density-weighted (PD) fat-saturated turbo spin echo (TSE) images, T1-weighted TSE images, T2-weighted TSE images, and contrast-enhanced T1-weighted fat-saturated TSE images (technical parameters are summarized in Supplement table 2). Contrast media (gadoterate meglumine, Dotarem, Guerbet) were administered according to the manufacturer’s recommendation at a dose of 0.2 mL/kg (0.1 mmol/kg) body weight as an intravenous bolus injection.

### Image analysis

Image analysis was performed on a commercially available workstation using Visage software (Visage 7.1, Pro Medicus Inc) by one radiology resident (after one semester of nearly exclusive training in musculoskeletal radiology) and two board-certified radiologists independently (8 years and 6 years of experience, respectively). All radiologists were blinded to clinical data.

The MRI dataset was analyzed twice by all 3 readers: in the first reading session (so-called unenhanced MRI), only MR images without contrast medium were included. In a second reading (so-called enhanced MRI), all imaging sequences (including post-contrast images) were included. A minimum time interval of 2 weeks was set between the two reading sessions to prevent recall bias.

All unenhanced and post-contrast MR images were systematically scored according to the following scoring systems:

The JAMRIS (juvenile arthritis MRI scoring) system encompasses synovial thickness, cartilage lesion, bone erosion, and bone marrow change (Supplement table 3) [13].

Modified IPSG (international prophylaxis study group) classification includes synovial thickness, cartilage lesion, bone erosion, subchondral cysts, and joint effusion (Supplement table 4) [27]. Synovia is hyperintense on non-enhanced T2- and PD-weighted imaging and was measured accordingly [28].

### Statistical analysis

Statistical analysis was performed using SPSS software (version 26; IBM Corporation) and MedCalc software (version 20.104; MedCalc Software Ltd). Intra-class correlation coefficient (ICC) was calculated to assess the inter-reader-agreement between the three readers for the JAMRIS and modified IPSG scores. Those were classified as follows: ICC < 0.5 poor, between 0.5 and 0.75 moderate, between 0.75 and 0.9 good, and greater than 0.90 excellent [29].

**Fig. 2:**
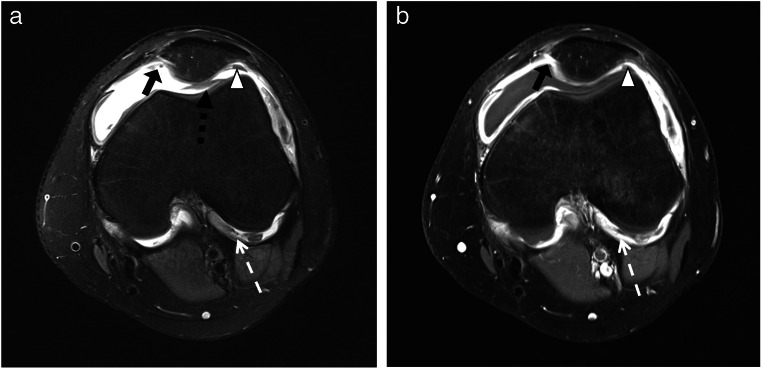
Twelve-year-old girl with extended oligoarthritis. Axial images demonstrating joint effusion and synovial membrane thickening, notably at the junction of the lateral condyle and the cruciate ligament (white dotted arrow), medial (black arrow), lateral (white arrowhead), and dorsal (black dotted arrow) to the patella, notably visible on image. **a** Proton-density-weighted fat-saturated sequence. **b** Contrast-enhanced T1-weighted turbo spin echo fat-saturated sequence

**Fig. 3:**
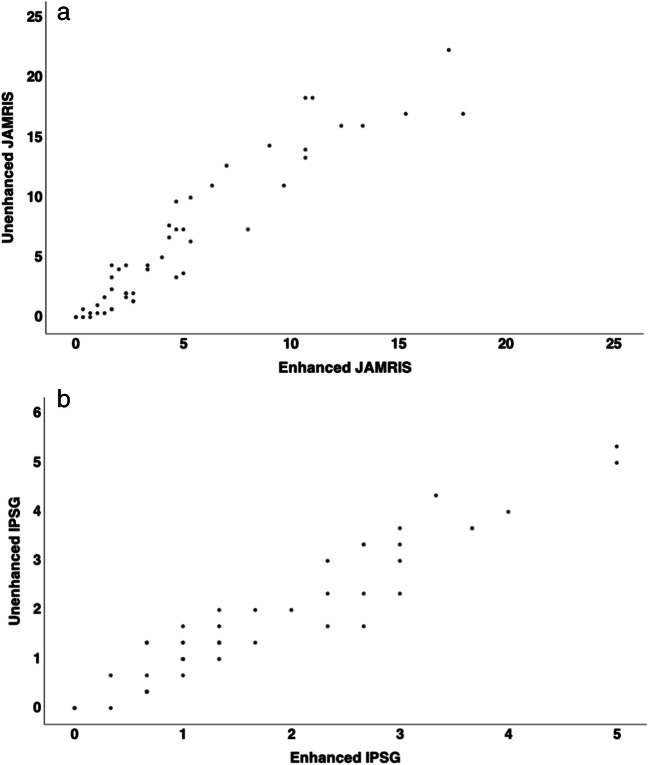
Scatter plots depicting the scores of the unenhanced images against the enhanced images for (**a**) JAMRIS, (**b**) IPSG

**Fig. 4:**
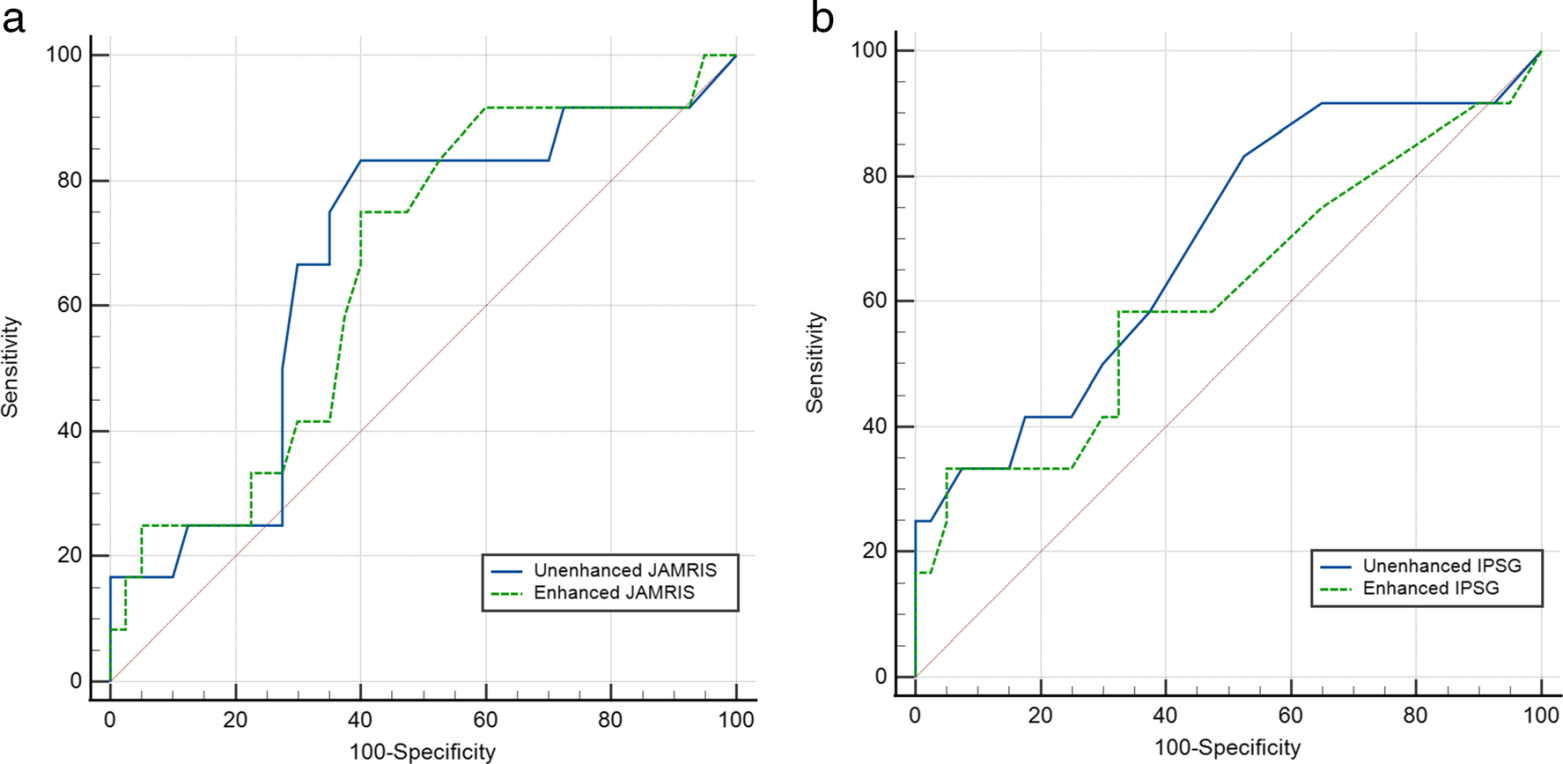
ROC curves of the unenhanced images against the enhanced images for (**a**) JAMRIS, (**b**) IPSG

JAMRIS and IPSG scores were applied to unenhanced and contrast-enhanced data sets. The arithmetic mean of MRI scores by all three readers was calculated, for any further analysis the arithmetic mean was used. Correlation analysis of both MRI scores was performed in comparison to clinically assessed disease activity using JADAS10. Statistical significance was calculated using the Pearson correlation coefficient (*r*) to show the correlation between the items, and the coefficient of determination (*R*^2^) was calculated to determine the proportion of the variation of the dependent variable. Scatter plots were used to illustrate the correlation between unenhanced and enhanced scoring. The diagnostic accuracy of MRI scores was determined using ROC curve analyses with comparison using DeLong’s test [30]. The cutoff for disease activity detected on MRI for both JAMRIS and IPSG was statistically determined using the Youden index based on the JADAS10. The cutoff for clinical disease activity was defined as a JADAS10 of ≤ 1.4 for oligoarthritis and ≤ 2.7 for polyarthritis [26]. A *p* value of < 0.05 was considered a significant difference (Figs. 2, 3, and 4).

## Results

### Disease activity

The mean JADAS10 was 2.26 ± 4.43. Eight out of the 27 patients had clinically active disease according to JADAS10 with all of those suffering from oligoarticular arthritis (see also Table 1). The mean JAMRIS score was 7.33 ± 10.19 for the unenhanced reading session, while the mean JAMRIS score was 5.59 ± 6.46 for the enhanced reading session. The mean score for the modified IPSG score was 1.72 ± 1.36 for the unenhanced and 1.64 ± 1.25 for the enhanced reading session.

### Intra-reader agreement

The intra-reader agreement was performed between unenhanced (first reading session) and enhanced (second reading session) MR images and determined by intra-class correlation for the three readers. The intra-reader agreement was good to very good for the JAMRIS (0.85 95% CI 0.81–0.88, 0.87 95% CI 0.83–0.89, and 0.96 95% CI 0.92–0.98) and good to very good for the IPSG score (0.76 95% CI 0.62–0.86, 0.86 95% CI 0.77–0.92, and 0.92 95% CI 0.86–0.96).

### Inter-reader agreement

Inter-reader agreement on scoring contrast-enhanced and unenhanced images was determined by intra-class correlation for the three readers [31]. Inter-reader agreement was good for the JAMRIS (0.83, 95% CI 0.75–0.89, and 0.82, 95% CI 0.74–0.89) and moderate for the IPSG score (0.65, 95% CI 0.51–0.76, and 0.62, 95% CI 0.48–0.75), unenhanced and contrast-enhanced respectively. ICC for synovial thickening applying JAMRIS and measured in multiple standardized locations was 0.83 for the unenhanced and 0.82 for the enhanced sequences, while ICC for synovial thickening using IPSG measured in the location with the maximal diameter was 0.53 for the unenhanced and 0.48 for the enhanced sequences. Unenhanced and enhanced MRI scores for IPSG were moderate (0.65, 95% CI 0.51–0.76, and 0.62, 95% CI 0.48–0.75) and high for JAMRIS (0.83, 95% CI 0.75–0.89, and 0.82, 95% CI 0.74–0.89).

### Correlation between unenhanced MRI and contrast-enhanced MRI

When using contrast-enhanced MRI as a reference standard, Pearson’s correlation coefficients between the unenhanced MRI and the enhanced MRI examination were high for both MRI scoring systems: JAMRIS (*r* = 0.97, *R*^2^ = 0.93, *p* < 0.01) and IPSG (*r* = 0.95, *R*^2^ = 0.91, *p* < 0.01). Pearson’s correlation between synovial thickening and joint effusion according to the modified IPSG score was moderate for both unenhanced (*r* = 0.47, *R*^2^ = 0.22, *p* < 0.01) and enhanced MRI (*r* = 0.56, *R*^2^ = 0.32, *p* < 0.01). Figure 1 demonstrates that synovial thickening involving the area around the cruciate ligaments can be well delineated not only on contrast-enhanced but also on unenhanced sequences.

### Correlation and diagnostic accuracy of MRI datasets when using the clinical JADAS10 as reference standard

When using the JADAS10 as a reference standard, statistical analysis evaluating MRI scores both before and after contrast agent administration showed an AUC of 0.68 (95% CI lower bound = 0.51, higher bound = 0.85), a sensitivity of 0.83, and a specificity of 0.61 for the JAMRIS acquired on unenhanced sequences. An AUC of 0.66 (95% CI lower bound = 0.49, higher bound = 0.82), a sensitivity of 0.75, and a specificity of 0.60 were detected for contrast-enhanced MRI. When comparing both JAMRIS AUC via DeLong’s test, the difference between areas was 0.02 with a standard error of 0.03 (*p* = 0.7). In the IPSG score, similar AUC were found in the ROC curve analysis for both unenhanced versus enhanced MRI examinations (AUC = 0.68, 95% CI lower bound = 0.50, higher bound = 0.86 vs. AUC = 0.61, 95% CI lower bound = 0.41, higher bound = 0.81). The difference between areas was 0.08 with a standard error of 0.04 (*p* = 0.07) via DeLong’s test when comparing the AUC of unenhanced and enhanced IPSG scores (Table 2). Statistically determined cutoff values using the Youden index when calculating the accuracy with the clinical JADAS10 were 4.17 for unenhanced and 3.76 for enhanced JAMRIS and 1.17 for the unenhanced and 1.5 for enhanced IPSG.

**Table 2:** AUC, sensitivity, and specificity of the MRI scores in comparison to the clinical JADAS10

	AUC	CI 95% lower bound	CI 95% higher bound	Sensitivity	Specificity
JAMRIS-enhanced MRI score	0.66	0.49	0.83	0.75	0.60
JAMRIS-unenhanced MRI score	0.68	0.51	0.85	0.83	0.61
JAMRIS-enhanced MRI synovial thickening	0.64	0.44	0.84	0.33	0.98
JAMRIS-unenhanced MRI synovial thickening	0.68	0.51	0.86	0.92	0.42
IPSG-enhanced MRI score	0.61	0.41	0.81	0.58	0.68
IPSG-unenhanced MRI Score	0.68	0.50	0.86	0.83	0.46
IPSG-enhanced MRI synovial thickening	0.63	0.42	0.85	0.50	0.85
IPSG-unenhanced MRI synovial thickening	0.67	0.49	0.87	0.50	0.83
IPSG-enhanced MRI joint effusion	0.51	0.31	0.71	0.17	1.00
IPSG-unenhanced MRI joint effusion	0.51	0.31	0.71	0.17	1.00

## Discussion

Both contrast-enhanced and unenhanced MRI of the knee were able to detect disease activity in JIA patients with similar accuracy when using standardized clinical reporting via JADAS10 as the gold standard. The accuracy of standardized reporting systems scored on unenhanced and enhanced MRI was not significantly different and therefore comparable with an AUC of 0.68 and 0.66 for JAMRIS (*p* = 0.7) and for IPSG of 0.68 and 0.61 (*p* = 0.07), respectively.

Inter-reader agreement for three readers was good for total JAMRIS (ICC: 0.82–0.83) and moderate for the IPSG score (ICC: 0.62–0.65). For individual items, Hemke et al [32] reported moderate to excellent ICC for JAMRIS (ICC: 0.55–0.95) and IPSG (0.57–0.94). Comparing ICC for MRI scores, our ICC for the total IPSG score was lower than for the JAMRIS. One reason could be different increments in comparable items for both scores such as bone erosion. JAMRIS is evaluated according to the involvement of total bone volume, whereas the modified IPSG score uses the total number of bone erosions with a maximum score in case of 2 or more bone erosions. Similarly, ICC was higher for synovial thickening using JAMRIS measured in multiple standardized locations compared to a lower ICC for synovial thickening when using IPSG based only on the location with the maximal synovial diameter. Furthermore, the IPSG score, initially developed for patients with hemophilic arthritis, is more extensive and includes subchondral cysts as a parameter which is not part of the JAMRIS. Nonetheless, the good intra- and inter-reader agreement for JAMRIS indicates relatively good applicability of the scoring system, even for more inexperienced readers. Therefore, it should be reasonable to have only one reader score the JAMRIS score for each patient in a clinical context.

The cutoff value for disease activity that was statistically determined using Youden’s index was 4.17 for unenhanced JAMRIS. In comparison, the cutoff value was slightly lower for the enhanced JAMRIS score of 3.76. However, this difference in cutoff values to determine disease activity in JIA may be regarded as negligible for readers, since JAMRIS uses integral scoring. For both enhanced and unenhanced JAMRIS, the cutoff value rounds up to a scoring of ≥ 4 in our study.

MRI of the knee in combination with standardized reporting has been proven to be a responsive outcome measure to monitor disease activity in JIA patients under anti-inflammatory medication [33]. The use of contrast media has been subject to discussions over the last years since intravenous administration of gadolinium-based contrast agents is associated with increased costs and risks [19]. One of the long-known risks is nephrogenic systemic fibrosis, which is a very rare complication that occurs in patients with impaired kidney function. Another recently described complication is the possible intracranial accumulation of gadolinium with unknown long-term consequences [20–22]. Therefore, application and especially repetitive application of MR contrast agents in children and adolescents must be well justified and should, if possible, be omitted.

Advances in MRI hard- and software development and the resulting improved image quality as well as image interpretation using artificial intelligence programs will likely help to better assess unenhanced images. This study contributes to the re-evaluation of the need and benefit of contrast agents in monitoring the disease activity of JIA. Hemke et al [15] reported that the reliability of the JAMRIS score for the assessment of synovial thickening decreases when omitting contrast agents in MRI examinations of the knee at 1 Tesla. Based on our data, no significant difference in diagnostic accuracy when using enhanced or unenhanced images could be found when using the clinical JADAS10 as a reference. In our study, we not only compared unenhanced and enhanced images but also included PD-weighted MRI, so it is possible to hypothesize that the accuracy of unenhanced images can be increased by additional evaluation of PD-weighted sequences. Furthermore, the higher magnetic field strength of 3 Tesla used in our study might lead to improved spatial resolution compared to a magnetic field strength of 1 Tesla used by Hemke et al [12]. Nevertheless, a recent review published on behalf of the European Society of Musculoskeletal Radiology (ESSR) arthritis subcommittee and the European Society of Paediatric Radiology (ESPR) indicated that diagnostic accuracy of unenhanced MRI is limited compared to contrast-enhanced MRI due to the reduced ability to evaluate synovitis [12]. In this article, recommendations are based on the literature review (among others: Hemke et al [15]) and/or expert opinion. To our knowledge, this is the first study demonstrating equal accuracy for the detection of active disease in JIA patients in unenhanced and enhanced 3 Tesla MRI [15].

A challenge in contrast agent application for the detection of disease activity of JIA is the standardization of image acquisition delay after administration. The synovial border is blurred by the process of diffusion of the contrast agent into the synovial fluid. Significant differences in synovial thickness measurements have been found dependent on the acquisition time of post-contrast images [17, 18]. Mean scores for synovial thickness and enhancement were reported significantly higher when based on late post-contrast images as compared to early post-contrast images in recent literature by Barendregt et al [34] and Rieter et al [35]: standardization of post-contrast image acquisition timing might thus be necessary to achieve reproducible results. Our results show unenhanced MRI to have similar accuracy for synovial measurement, rendering timing problems during image acquisition obsolete.

A recent study suggested that synovitis may often present without joint effusion, although joint effusion is routinely measured in the sonographic assessment of disease activity [36]. In our study, we could detect a moderate correlation between synovial thickening and joint effusion for unenhanced and enhanced images evaluated by the IPSG score. The JAMRIS score does not record joint effusion. Further studies are necessary to evaluate the role of joint effusion in JIA and to develop a standardized assessment of joint effusion in JIA.

Images acquired at 3 Tesla produce higher image quality resulting in an increase in diagnostic accuracy. Spatial resolution improves with increasing magnetic field strength, which might be a key factor in rendering a reliable assessment of synovial thickening possible in unenhanced images in our study (Table 2). Higher magnetic field strength might therefore help to better assess disease activity in unenhanced images with similar accuracy when compared to contrast-enhanced images [37, 38].

The prolonged duration of a contrast-enhanced MRI examination is another argument against the application of contrast agents, as younger patients are prone to movements during longer examination times. Longer examination times might necessitate sedation, which itself poses a potential health risk [19, 39].

Diffusion-weighted imaging (DWI) has been evaluated as a non-invasive parameter to detect disease activity in patients with JIA [40, 41]. In a recent study, high accuracy for the detection of arthritis and agreement with contrast-enhanced MRI has been found for DWI. The authors proposed the replacement of contrast-enhanced sequences with DWI [36]. Advanced quantitative MRI techniques, such as T2-mapping and T1 rho mapping, might play an important role in the future for the evaluation of inflammatory changes in the knee, but these sequences are not yet part of routine imaging [42]. These might also contribute to reliable imaging of JIA without contrast agents.

Another imaging technique proven valuable for the detection of synovitis and enthesitis is ultrasound [43]. The advantages of US over MRI are lower costs, short examination time, easy accessibility, and the ability to depict soft tissue inflammation without the use of contrast agents [44], even though contrast media might further improve the evaluation of subclinical synovitis [45]. However, ultrasound US has limited reproducibility since no standardized imaging or reporting protocols exist. Deep joint spaces cannot be assessed due to acoustic shadowing from overlying bones and this method is limited in large or obese children. Cruciate ligament synovial involvement, a structure of deep joint space, has been shown to be specific for JIA and improves discrimination between JIA and unaffected children. This discrimination is possible only with MRI but not with US [14], rendering MRI an indispensable diagnostic tool in JIA. Another great advantage is the possibility to examine the entire joint.

Our study has limitations. Not all MRI examinations of the knee were conducted with a dedicated knee coil. In patients in need of an MRI of both knees, this examination was carried out in some patients in one single examination using a multi-channel body matrix. However, the use of surface coils for MR imaging of the knee might be nonetheless sufficient under certain circumstances and can produce images of acceptable quality [46–48]. Due to the small number of knee examinations acquired using a dedicated knee coil in our study, no clear conclusion can be drawn and further investigation is needed. We used the Youden index to calculate specificity and sensitivity. Since the cutoff point is determined via statistical analysis and disregards clinical factors, the specificity was higher than the sensitivity.

Another limitation is the small number of patients involved as this was a single-center study with a retrospective study design. In addition, for most patients, no follow-up MRI was available to evaluate and correlate with clinical therapy response. However, this is the first study to show unenhanced MR images acquired on a 3T MRI to detect synovial thickening of the knee joint as reliably as on enhanced MR images.

## Conclusion

We could show that unenhanced MRI, using an augmented protocol comprising PD-weighted sequences, can detect disease activity in patients with JIA with equally high accuracy compared to contrast-enhanced MRI.

## Supplementary Information

ESM 1 (DOCX 58 kb)

## Declarations

### Guarantor

The scientific guarantor of this publication is Prof. Dr. Diane Renz.

### Statistics and biometry

No complex statistical methods were necessary for this paper.

### Methodology

• retrospective

• diagnostic or prognostic study / observational

• performed at one institution

## Abbreviations

AUC: Area under the curve
CI: Confidence interval
DWI: Diffusion-weighted imaging
ICC: Intra-class correlation coefficient
IPSG: International prophylaxis study group
JADAS: Juvenile arthritis disease activity score
JAMRIS: Juvenile arthritis MRI scoring
JIA: Juvenile idiopathic arthritis
MRI: Magnetic resonance imaging
PD: Proton density
ROC: Receiver operating characteristics
TSE: Turbo spin echo
US: Ultrasound
VAS: Visual analogue scale

## References

[1] Petty RE, Southwood TR, Manners P (2004). International League of Associations for Rheumatology classification of juvenile idiopathic arthritis: second revision, Edmonton, 2001. J Rheumatol.

[2] Ravelli A, Martini A (2007). Juvenile idiopathic arthritis. Lancet.

[3] Hemke R, Doria AS, Tzaribachev N, Maas M, van der Heijde DMFM, van Rossum MAJ (2014). Selecting magnetic resonance imaging (MRI) outcome measures for juvenile idiopathic arthritis (JIA) clinical trials: first report of the MRI in JIA special interest group. J Rheumatol.

[4] Palmisani E, Solari N, Magni-Manzoni S (2006). Correlation between juvenile idiopathic arthritis activity and damage measures in early, advanced, and longstanding disease. Arthritis Rheum.

[5] Oen K, Reed M, Malleson PN (2003). Radiologic outcome and its relationship to functional disability in juvenile rheumatoid arthritis. J Rheumatol.

[6] Oen K, Duffy CM, Tse SML (2010). Early outcomes and improvement of patients with juvenile idiopathic arthritis enrolled in a Canadian multicenter inception cohort. Arthritis Care Res.

[7] Albers HM, Wessels JAM, van der Straaten RJHM (2009). Time to treatment as an important factor for the response to methotrexate in juvenile idiopathic arthritis. Arthritis Rheum.

[8] Vilca I, Munitis PG, Pistorio A (2010). Predictors of poor response to methotrexate in polyarticular-course juvenile idiopathic arthritis: analysis of the PRINTO methotrexate trial. Ann Rheum Dis.

[9] Martini A, Lovell DJ (2010). Juvenile idiopathic arthritis: state of the art and future perspectives. Ann Rheum Dis.

[10] Alves TI, Girish G, Kalume Brigido M, Jacobson JA (2016). US of the knee: scanning techniques, pitfalls, and pathologic conditions. Radiographics.

[11] Gylys-Morin VM, Graham TB, Blebea JS (2001). Knee in early juvenile rheumatoid arthritis: MR imaging findings. Radiology.

[12] Hemke R, Herregods N, Jaremko JL (2020). Imaging assessment of children presenting with suspected or known juvenile idiopathic arthritis: ESSR-ESPR points to consider. Eur Radiol.

[13] Hemke R, van Rossum MAJ, van Veenendaal M (2013). Reliability and responsiveness of the juvenile arthritis MRI scoring (JAMRIS) system for the knee. Eur Radiol.

[14] Nusman CM, Hemke R, Benninga MA (2016). Contrast-enhanced MRI of the knee in children unaffected by clinical arthritis compared to clinically active juvenile idiopathic arthritis patients. Eur Radiol.

[15] Hemke R, Kuijpers TW, van den Berg JM (2013). The diagnostic accuracy of unenhanced MRI in the assessment of joint abnormalities in juvenile idiopathic arthritis. Eur Radiol.

[16] Hemke R, Nusman CM, van der Heijde DMFM (2015). Frequency of joint involvement in juvenile idiopathic arthritis during a 5-year follow-up of newly diagnosed patients: implications for MR imaging as outcome measure. Rheumatol Int.

[17] Michalski E, Ostrowska M, Gietka P, Sudoł-Szopińska I (2020). Magnetic resonance imaging of the knee joint in juvenile idiopathic arthritis. Reumatologia.

[18] Hemke R, Kuijpers TW, Nusman CM (2015). Contrast-enhanced MRI features in the early diagnosis of juvenile idiopathic arthritis. Eur Radiol.

[19] Jaimes C, Murcia DJ, Miguel K, DeFuria C, Sagar P, Gee MS (2018). Identification of quality improvement areas in pediatric MRI from analysis of patient safety reports. Pediatr Radiol.

[20] McDonald RJ, McDonald JS, Kallmes DF (2015). Intracranial gadolinium deposition after contrast-enhanced MR imaging. Radiology.

[21] Rozenfeld MN, Podberesky DJ (2018). Gadolinium-based contrast agents in children. Pediatr Radiol.

[22] Renz DM, Kümpel S, Böttcher J et al (2018) Comparison of unenhanced T1-weighted signal intensities within the dentate nucleus and the globus pallidus after serial applications of gadopentetate dimeglumine versus gadobutrol in a pediatric population. Invest Radiol 53(2):119–127 10.1097/RLI.0000000000000419 28976476

[23] Kozak BM, Jaimes C, Kirsch J, Gee MS (2020). MRI techniques to decrease imaging times in children. Radiographics.

[24] Consolaro A, Giancane G, Schiappapietra B (2016). Clinical outcome measures in juvenile idiopathic arthritis. Pediatr Rheumatol Online J.

[25] Consolaro A, Bracciolini G, Ruperto N (2012). Remission, minimal disease activity, and acceptable symptom state in juvenile idiopathic arthritis: defining criteria based on the juvenile arthritis disease activity score. Arthritis Rheum.

[26] Trincianti C, van Dijkhuizen EHP, Alongi A (2021). Definition and validation of the American College of Rheumatology 2021 Juvenile Arthritis Disease Activity Score Cutoffs for Disease Activity States in Juvenile Idiopathic Arthritis. Arthritis Rheum.

[27] Lundin B, Manco-Johnson ML, Ignas DM (2012). An MRI scale for assessment of haemophilic arthropathy from the International Prophylaxis Study Group. Haemophilia.

[28] Burke CJ, Alizai H, Beltran LS, Regatte RR (2019). MRI of synovitis and joint fluid. J Magn Reson Imaging.

[29] Koo TK, Li MY (2016). A guideline of selecting and reporting intraclass correlation coefficients for reliability research. J Chiropr Med.

[30] DeLong ER, DeLong DM, Clarke-Pearson DL (1988). Comparing the areas under two or more correlated receiver operating characteristic curves: a nonparametric approach. Biometrics.

[31] Shrout PE, Fleiss JL (1979). Intraclass correlations: uses in assessing rater reliability. Psychol Bull.

[32] Hemke R, Tzaribachev N, Nusman CM, van Rossum MAJ, Maas M, Doria AS (2017). Magnetic resonance imaging (MRI) of the knee as an outcome measure in juvenile idiopathic arthritis: an OMERACT reliability study on MRI scales. J Rheumatol.

[33] Hemke R, van Veenendaal M, van den Berg JM (2014). One-year followup study on clinical findings and changes in magnetic resonance imaging-based disease activity scores in juvenile idiopathic arthritis. J Rheumatol.

[34] Barendregt AM, van Gulik EC, Groot PFC (2019). Prolonged time between intravenous contrast administration and image acquisition results in increased synovial thickness at magnetic resonance imaging in patients with juvenile idiopathic arthritis. Pediatr Radiol.

[35] Rieter JFMM, de Horatio LT, Nusman CM (2016). The many shades of enhancement: timing of post-gadolinium images strongly influences the scoring of juvenile idiopathic arthritis wrist involvement on MRI. Pediatr Radiol.

[36] Roemer FW, Kassim Javaid M, Guermazi A et al (2010) Anatomical distribution of synovitis in knee osteoarthritis and its association with joint effusion assessed on non-enhanced and contrast-enhanced MRI. Osteoarthritis Cartilage 18(10):1269–1274 10.1016/j.joca.2010.07.008 20691796

[37] Wong S, Steinbach L, Zhao J, Stehling C, Ma CB, Link TM (2009) Comparative study of imaging at 3.0 T versus 1.5 T of the knee. Skeletal Radiol 38(8):761–769 10.1007/s00256-009-0683-0 PMC2704948 19350234

[38] Cheng Q, Zhao F-C (2018). Comparison of 1.5- and 3.0-T magnetic resonance imaging for evaluating lesions of the knee: a systematic review and meta-analysis (PRISMA-compliant article). Medicine (Baltimore).

[39] Jaimes C, Gee MS (2016). Strategies to minimize sedation in pediatric body magnetic resonance imaging. Pediatr Radiol.

[40] Barendregt AM, Mazzoli V, van Gulik EC (2020). Juvenile idiopathic arthritis: diffusion-weighted MRI in the assessment of arthritis in the knee. Radiology.

[41] Sauer A, Li M, Holl-Wieden A, Pabst T, Neubauer H (2017). Readout-segmented multi-shot diffusion-weighted MRI of the knee joint in patients with juvenile idiopathic arthritis. Pediatr Rheumatol Online J.

[42] Barendregt AM, Mazzoli V, van den Berg JM (2020). T1ρ-mapping for assessing knee joint cartilage in children with juvenile idiopathic arthritis - feasibility and repeatability. Pediatr Radiol.

[43] Chauvin NA, Doria AS (2017). Ultrasound imaging of synovial inflammation in juvenile idiopathic arthritis. Pediatr Radiol.

[44] Magni-Manzoni S (2016). Ultrasound in juvenile idiopathic arthritis. Pediatr Rheumatol Online J.

[45] Mouterde G, Carotti M, D’Agostino MA (2009). Échographie de contraste et pathologie ostéo-articulaire. J Radiol.

[46] Kogan F, Levine E, Chaudhari AS (2018). Simultaneous bilateral-knee MR imaging. Magn Reson Med.

[47] van den Steen M, de Maeseneer M, Hoste M, Vanderdood K, de Ridder F, Osteaux M (2003). Comparison of surface coil and knee coil for evaluation of the patellar cartilage by MR imaging. Eur J Radiol.

[48] Aarvold A, Pope A, Sakthivel VK, Ayer RV (2014) MRI performed on dedicated knee coils is inaccurate for the measurement of tibial tubercle trochlear groove distance. Skeletal Radiol 43(3):345–349 10.1007/s00256-013-1790-5 24362937

