# Supplementary material for: Juvenile idiopathic arthritis of the knee: is contrast needed to score disease activity when using an augmented MRI protocol comprising PD-weighted sequences?
Source: Eur Radiol. 2022 Dec 6;33(5):3775–84. doi: 10.1007/s00330-022-09292-3 (PMC10121492; doi:10.1007/s00330-022-09292-3)
Supplement: Supplementary file 1 — (DOCX 58 kb) [file 330_2022_9292_MOESM1_ESM.docx]

**Supplement table**

**Supplement table 1:** JADAS10 (Juvenile arthritis disease activity score) [24]

| Physician global assessment | 0–10-cm VAS |
| --- | --- |
| Parent/patient global  assessment | 0–10-cm VAS |
| Active joint count | Simple count, 0–10 (any count higher than 10 is cutoff at a max of 10) |
| Acute-phase reactant (range) | Normalized ESR (0–10) |
| Score range (total) | 0-40 |

**Supplement table 2:** Sequence protocol used in our study

| Sequences | FoV | Slice thickness | Slice spacing | TR | TE | Matrix | Bandwidth | Flip angle |
| --- | --- | --- | --- | --- | --- | --- | --- | --- |
| PD tse fs tra | 280 x 280 | 3 mm | 10 % | 5740 ms | 39 ms | 512 x 384 | 181 Hz/Px | 150° |
| T1 tse sag | 200 x 200 | 3 mm | 10 % | 734 ms | 11 ms | 448 x 314 | 260 Hz/Px | 150° |
| PD tse fs sag | 200 x 200 | 3 mm | 10 % | 2530 ms | 33 ms | 448 x 314 | 199 Hz/Px | 150° |
| T2 tse tra | 280 x 280 | 3 mm | 10 % | 3410 ms | 99 ms | 512 x 256 | 257 Hz/Px | 140° |
| T1 tse fs tra enhanced | 300 x 300 | 3 mm | 10 % | 826 ms | 11 ms | 448 x 314 | 260 Hz/Px | 150° |
| T1 tse fs sag | 200 x 200 | 3 mm | 10 % | 701 ms | 11 ms | 448 x 314 | 260 Hz/Px | 150° |

FoV=Field of view; TR= repetition time; TE=echo time, PD=proton density, tse=turbo spin echo, fs= fat-saturated, sag= sagittal, tra=transversal

**Supplement table 3:** JAMRIS (Juvenile arthritis MRI scoring) system [13]

| Synovial thickening (max. thickness) | 0-2mm = 1, >2-4mm = 2, >4mm = 3 |
| --- | --- |
| Cartilage lesion (involvement of cartilage surface area) | None = 1, <10% = 2, 10-25% = 3, >25% = 4 |
| Bone marrow change (involvement of bone volume) | None = 1, <10% = 2, 10-25% = 3, >25% = 4 |
| Bone erosion score (involvement of bone volume) | None = 1, <10% = 2, 10-25% = 3, >25% = 4 |

**Supplement table 4:** Modified IPSG (International prophylaxis study group) classification [26, 28, 45],

| Synovial thickening (maximal thickness in location with maximal Score) | 0 = none, >2-3mm = 1, >3-5mm = 2, >5mm = 3 |
| --- | --- |
| Joint effusion | >5-7mm = 1, >7-15mm = 2, > 15mm = 3 |
| Bone erosion (maximum of 2 in total) | any bone erosion = 1, >50% = +1 |
| Subchondral cysts (maximum of 2 in total) | at least 1 subchondral cyst = 1, subchondral cysts in 2 bones = +1 |
| Cartilage lesions/loss | any cartilage lesion = 1, >50% = +1, full thickness loss= 1, full thickness loss >50% = +1 |
